# Caregiver Psychopathology, Resilience, and Their Associations with Social-Emotional Challenges of Young Children Affected by Armed Conflict in Colombia

**DOI:** 10.1007/s10578-024-01787-y

**Published:** 2024-11-22

**Authors:** Sascha Hein, Liliana A. Ponguta, José M. Flores, Amalia Londoño Tobón, Isaac N. S. Johnson, Julie Larran, Ana M. Ortiz Hoyos, Oscar Gómez, Lina M. González Ballesteros, Camila A. Castellanos Roncancio, James F. Leckman

**Affiliations:** 1 Department of Education and Psychology, Freie Universität Berlin, Fabeckstraße 35, 14195 Berlin, Germany; 2 Yale Child Study Center, New Haven, CT USA; 3 MedStar Georgetown University Hospital, Washington, DC USA; 4 Fundación Saldarriaga Concha, Bogotá, Colombia; 5 Pontificia Universidad Javeriana, Bogotá, Colombia

**Keywords:** Anxiety, Colombia, Caregiver, Depression, Early childhood

## Abstract

We examined the social and emotional challenges (SEC) of young children and the mental health of their caregivers in areas affected by armed conflict and displacement in Colombia. Anxiety, depression, and PTSD symptoms were assessed in 1,133 caregivers. Caregivers also reported on the SEC of their children aged 21–53 months. A subsample of 487 caregivers (43%) completed a resilience self-report measure. Caregivers reported substantial levels of psychopathology ranging from 12.84% of caregivers with elevated anxiety levels, 24.5% who scored above the cutoff on the PTSD measure, and 44.7% who screened positive for depression. Correlations (*r*) between child SEC and caregiver psychopathology ranged from 0.15 to 0.33. The association between caregiver depression and child SEC was more substantial for boys (*B* = 0.56) than girls (*B* = 0.22). We also found that girls whose caregivers reported elevated levels of psychopathology and low levels of caregiver resilience received the highest ratings of caregiver-reported SEC. The importance of caregiver-child resilience-focused psychosocial support is discussed.

**Supplementary Information:**

The online version contains supplementary material available at 10.1007/s10578-024-01787-y.

## Introduction

Globally, multiple factors, such as socioeconomic and geopolitical stressors, underpin caregivers’ well-being, mental health, and resilience [43]. These stressors may prevent caregivers from providing adequate care to their children, which increases the risk of their children exhibiting cognitive, behavioral, and emotional adjustment difficulties/issues [29]. It is imperative to support caregivers in building their resilience and mental health to provide a safe and nurturing environment where their children can grow into healthy, functioning, and well-adjusted adults. Understanding and supporting caregivers' mental health and resilience in conflict settings is critical to promoting responsive caregiving and ensuring an effective humanitarian health response for conflict-affected  caregivers and children (e.g., [2]). Colombia offers a unique context to examine the impacts of stress and conflict on caregiver psychopathology and resilience and their relation to early childhood development (ECD).

For over 50 years, Colombia has been affected by civil conflict, which has had lingering effects on caregivers and children. Many young children are or have been exposed to multiple developmental risks, such as poverty, civil conflict, and domestic violence [26]. A cross-sectional study of 7–11-year-old children in an region of Colombia affected by armed conflict demonstrated that 6.5% of displaced children met the criteria for an anxiety disorder compared to 1.8% of non-displaced children. Among displaced children, 13.2% scored high for post-traumatic stress compared to 6.6% of non-displaced children [14]. These data  align with elevated mental health concerns reported among general adults in the 2015 Colombian National Mental Health Survey (NMHS; [11]). A recent study found PTSD prevalence rates of 12.3% and 11% for any mental health disorder (including depressive disorders, dysthymia, mania, generalized anxiety disorder, panic disorder, social phobia, and suicidal ideation)   in a sub-sample of displaced adolescents [26].  Additionally,  using the 2015 NMHS data, Tamayo Martínez et al. [38] found a 15.9% lifetime prevalence of any psychiatric disorder in displaced adults. Thus, promoting caregiver mental health is a vital priority, and there is a need for empirical evidence on the prevalence of and factors contributing to the mental health and well-being of Colombian caregivers, particularly for conflict-affected and internally displaced people [39].

In addition to research on caregiver mental health and child adjustment in Colombian families, emerging research suggests a link between the two. For instance, a study by Cuartas [9] investigated the effects of community violent crime on maternal parenting behavior. Exposure to violent crimes predicted a higher probability of maternal corporal disciplinary measures such as hitting children with objects [9]. Recent work by Cuartas and Leventhal [10] utilized NMHS data to demonstrate that community violent crime was associated with mental health conditions in children aged 7–11. Furthermore, caregiver mental health was found to moderate the association between exposure to an incident of community violent crime and children's mental health. Flink et al. [13] investigated the association between forced internal displacement in Colombia and the mental health of children between 1.5 and 5 years old. The authors found that displaced children met borderline cut-off scores more often than non-displaced children, even after adjustment for socio-demographic factors. Moreover, the authors found that family functioning and caregiver mental health were strongly and independently associated with the mental health of internally displaced children. These findings highlight the importance of caregiver mental health as a potential risk factor.

Among the protective factors against adversity, resilience has emerged as a critical individual and community characteristic. Resilience is a concept investigated in recent decades as an underlying mechanism of coping strategies [22]. Studies have recently highlighted the interplay between cultural and political contexts in Colombia and their impact on women’s well-being and resilience [47]. In Colombia, recent studies have also explored the impact of psychosocial programs focused on caregivers of young children on parental outcomes, including resilience [16, 21]. The data suggest that resilience is a concept interlinked with the sociocultural and geopolitical context, individual adaptive and coping mechanisms, and therefore integral to the impact of conflict and adversity on caregiver well-being and, consequently, children’s experiences and developmental outcomes.

In addition to the direct links between caregiver mental health and child adjustment, research conducted in high income countries suggests that these links are moderated by child sex. For instance, in a longitudinal study with 198 mother–child dyads in a large metropolitan area in the U.S., maternal PTSD symptoms have been found to predict problem behaviors in 18-month-old toddlers (assessed using the CBCL/1.5–5) for boys but not for girls [28]. Similarly, another study with younger children found boys to be more vulnerable to the effects of maternal psychopathology [23]. Letourneau et al. [23], studied adverse childhood experiences (ACEs) and depression, anxiety, and social support (3, 6, and 12 months postpartum) in 907 Canadian mothers and the effects on their children. The authors found evidence for sex-moderated associations such that the associations between maternal anxiety and depressive symptoms and child internalizing behavior (assessed at the age of 2 years using the CBCL) were stronger for boys than girls. Sex-specific effects of caregiver psychopathology have been documented, particularly the more significant effect of maternal depression on infant boys compared with girls [31].

In summary, except for a few studies using the Colombian NMHS data (e.g., [10]), there is a scarcity of research on caregiver mental health and its relationship to child adjustment, particularly in conflict affected countries and regions. The current available research from Colombia has focused on caregiver and child behavior and mental health of school-age children (e.g., children aged 7–11 years old; [10]) rather than young children (children aged 0–5).

### The Present Study

We utilized data from a large-scale epidemiological survey on adult mental health in Colombia to answer the following question: What are the links between caregiver psychopathology and social-emotional challenges (SEC) of children between the ages of 21 and 53 months in Colombia? Four specific aims guided this research.

First, we aimed to quantify levels of anxiety, depression, and symptoms of post-traumatic stress (henceforth, collectively referred to as psychopathology) among Colombian caregivers of young children, along with rates of comorbidities of these conditions. At the time of the study, the caregivers lived in municipalities most exposed to the war and violence in Colombia and in which development is prioritized as per the territorially focused development plan (Programas de Desarrollo con Enfoque Territorial, or PDETs in Spanish). Children were enrolled in local Early Childhood Development (ECD) centers. PDETs are an initiative designed to promote sustainable development in regions of Colombia that have been most affected by armed conflict. These programs aim to address structural issues and inequalities by fostering economic growth, improving infrastructure, and enhancing social services in targeted territories. PDETs focus on participatory planning and involve local communities in the decision-making process to ensure that the development strategies are tailored to each area’s specific needs and conditions. By prioritizing the regions that suffered the most from conflict, PDETs seek to build peace and stability through inclusive development and the strengthening of local governance. As this was a descriptive aim, no specific hypotheses were formulated.

The second aim was to examine the associations between caregiver psychopathology and caregiver-reported social and emotional challenges (SEC) of their child. Based on prior research conducted in Colombia, showing direct links [13] and moderated associations [10], we expected to find small, statistically significant, and positive associations between caregiver psychopathology and child SEC (Hypothesis 1, H1).

Third, we examined interactions between caregiver psychopathology and child sex and their associations with caregiver-reported SEC of their child. Despite the inconsistent findings of previous studies reviewed above, we hypothesized  boys to be more vulnerable to the effects of caregiver psychopathology compared to girls (H2) [23].

Finally, to our knowledge, no study has examined caregiver resilience as a buffering factor in the relationship between caregiver psychopathology and child SEC in Colombia. Therefore, our fourth aim was to probe interactions between caregiver resilience and psychopathology that may predict child SEC. We hypothesized that children of caregivers with high levels of psychopathology and low levels of resilience exhibit higher levels of SEC when compared with children of caregivers with high levels of psychopathology but who reported high levels of resilience (H3).

## Method

### Sample

A total of 1133 caregivers (87.6% female; mean age = 29.39 years) from 14 municipalities in Colombia participated in this study. Caregivers included biological parents, grandparents, and other persons (e.g., close family members) responsible for caring for the child. Caregivers were asked to report on the child’s sex and other demographic characteristics. Participant characteristics are referenced in Table 1. This is a subsample of over 2000 caregivers who participated in an epidemiological survey and had available data on the measures used in this study. The survey data were collected from June to December 2015. The municipalities were selected because of their exposure to armed conflict through the direct presence of armed groups, geographic proximity to drug trafficking routes, or because they were receptor areas of displaced rural communities. The 14 municipalities included in the study are categorized as areas with high levels of violence by the Colombian government and international organizations. In Colombia, individuals have the option to register themselves as victims of the armed conflict with the government, which has implications for their security. Therefore, there is significant underreporting of this information. While Table 1 shows that 51.5% of the caregivers were not victims of forced displacement and 60% were not victims of armed conflict, these figures reflect the self-reported status of the participants. Living in a high-risk municipality was a critical inclusion criterion, regardless of whether the individuals were directly affected by forced displacement or armed conflict. The Instituto Colombiano de Bienestar Familiar (ICBF) selected the target population. The caregivers were eligible to participate in a resilience skills-building intervention designed and implemented by the Fundación Saldarriaga Concha (FSC) [16]. However, when the data for the present study were collected, the caregivers had not received any specific intervention. Participants were selected through a non-probabilistic convenience sampling strategy from a list of ECD centers and Community-Based Family Homes operated by ICBF, which also registers victims of the armed conflict.

**Table 1:** Sample characteristics

	*n*	% or *M* (*SD*)
*Child*		
Age (months)	1133	39.08 (8.99)
*Sex*		
Male	535	47.2
Female	580	51.2
Missing	18	1.6
*Birth order*		
First-born	1109	97.9
Second-born	24	2.1
*Disability (parent report)*		
Yes	389	34.3
No	687	60.6
Missing	57	5
*Victim of forced displacement*		
Yes	500	44.1
No	575	50.8
Missing	58	5.1
*Victim of armed conflict*		
Yes	231	20.4
No	845	74.6
Missing	57	5
*Municipality*		
Buenaventura	75	6.6
El Tambo	65	5.7
Guapi	77	6.8
Istmina	93	8.2
Maicao	28	2.5
Medellín	78	6.9
Necoclí	97	8.6
Pasto	65	5.7
San Vincente del Caguán	99	8.7
Sincelejo	80	7.1
Soledad	57	5.0
Tame	86	7.6
Tumaco	67	5.9
Turbo	166	14.7
*Caregiver*		
Age (years)	1041	29.39 (8.32)
Missing	92	8.12
*Sex*		
Male	38	3.4
Female	992	87.6
Missing	103	9.1
*Victim of forced displacement*		
Yes	471	41.6
No	584	51.5
Missing	78	6.9
*Victim of armed conflict*		
Yes	395	34.9
No	681	60.1
Missing	57	5.0

*N* = 1133. Child age ranged from 21 to 53 months. Caregiver age ranged from 16 to 75 years

### Measures

#### Caregiver-Reported Social-Emotional Challenges of Children: Ages and Stages Questionnaire—Social Emotional (ASQ-SE)

The Spanish version of the Ages and Stages Questionnaire: Social-Emotional [37] was used to measure caregiver-reported social and emotional development of their children. Some mothers (*n* = 53) had multiple children in the suitable age range and, thus, filled out one ASQ-SE survey according to the age of each child, resulting in a total number of 1186 observations (*n* = 211 24-month surveys; *n* = 429 36-month surveys; *n* = 546 48-month surveys). Two example items of the 24-month version are “When you leave, does your child remain upset and cry for more than an hour?” and “Does your child have trouble falling asleep at naptime or at night?” The surveys were scored according to the instructions provided in the ASQ-SE manual. In short, responses (most of the time; sometimes; rarely or never) were converted to numerical equivalents of 0, 5, and 10 for each item. All items were then summed up to a total score. When a parent marks an item as an area of concern, 5 points are added to the total score. Total scores exceeding the cutoff scores of 50, 59, and 70 for the 24-, 36- and 48-month versions respectively indicate a possible need for referral for further evaluation due to elevated SEC. The internal consistencies of the three ASQ-SE surveys were adequate (Cronbach’s α = 0.77, 0.80, and 0.81 for the 24-, 36-, and 48-month questionnaires respectively). In addition to the age-specific scores, one overall ASQ-SE score across the three age-specific versions (24, 36, and 48 months) was calculated by computing mean-centered scores for each age version divided by the pooled standard deviation.

#### Caregiver’s Depressed Mood and Anhedonia

The Whooley is a two-question case-finding instrument for depression that asks about depressed mood and anhedonia. It has a sensitivity of 96% (95% CI 90–99%) and specificity of 57% (95% CI 53–62%) when a positive answer to any of the two items is given [45]. The Whooley questions are recommended as a screening tool in the Colombian clinical practice guideline for depression [30]. For clarity and conciseness, we use the term caregiver depression in case of a positive screening (i.e., the answer to at least one of both questions was affirmative).

#### Caregiver Anxiety

The Hamilton Anxiety Scale (HAM-A) is a 14-item self-report measure developed as a scoring system for anxiety that fits well with clinical evaluations [20]. Anxiety severity is rated as mild if scores are ≤ 17, mild to moderate if scores range from 18 to 24, moderate to severe if scores range from 25 to 30, and very severe if scores > 30, in a 0 to 56 score range [20]. The HAM-A has been validated in Spanish, with results showing psychometric properties similar to those of the original version (Cronbach’s α = 0.89; Intraclass correlation coefficient = 0.92; Effect size [Sensitivity to change] = 1.36), being considered as a suitable tool for research and clinical use [25]. Internal consistency in our study was good (Cronbach’s α = 0.87).

#### Caregiver PTSD

The Post-Traumatic Stress Disorder Checklist—Civilian version (PCL-C) is a 17-item self-report measure of civilians’ response to traumatic experiences [46]. Total scores range from 17 to 85 and are based on the amount and severity of PTSD-related symptoms (symptom severity ranges from 1 = Not at all to 5 = Extremely). The cut-off score for possible PTSD is greater or equal to 30 (sensitivity = 82%; specificity = 76%) [44]. The PLC-C has been used as screening for PTSD in Colombia’s 2015 NMHS [38]. Internal consistency in our study was excellent (Cronbach’s α = 0.92).

#### Caregiver Resilience

The Connor–Davidson Resilience Scale (CD-RISC; [7]) comprises 25 items that measure five factors: personal competence, tolerance and strength, positiveness, control, and spiritual influences. Several studies have focused on Spanish-speaking populations and validated different versions of CD-RISC [8]. We evaluated the psychometric properties of the CD-RISC with a sample of 1075 caregivers who had completed the CD-RISC as part of their participation in the epidemiological survey. In this sample, a latent variable measurement model with the 10-item version of the CD-RISC [3] in which the uniqueness of items 7 and 8 was freely estimated yielded adequate fit to the data (RMSEA = 0.048, 90% CI 0.039, 0.058; CFI = 0.930, TLI = 0.908; SRMR = 0.038). The reliability was acceptable (Cronbach’s α = 0.75). Factor scores were saved from this model and used for the analyses. In this study, CD-RISC data were available for 487 of 1133 caregivers (about 43%) who also had data on the other measures used in this study. Compared to caregivers who completed the CD-RISC, caregivers with missing CD-RISC data reported higher levels of anxiety (*M* = 9.94 vs. *M* = 8.88, *t*(1058) = −2.30, *p* = 0.022) and PTSD (*M* = 26.61 vs. *M* = 24.82, *t*(1013.12) = −2.94, *p* = 0.003), and were more likely to screen positive for depression (61.1% vs. 52.2%; χ^2^(1) = 8.60, *p* = 0.003).

### Procedure

The details of the data collection procedures are described in a recent study evaluating the 3Cs program for caregivers of young children affected by the armed conflict in Colombia [16]. In brief, the data were gathered through in-personl individual interviews in the municipalities. During these interviews, trained personnel utilized an electronic data entry system on tablet devices to record the information. Data were uploaded from the tablets into a centralized REDCap data management system. The lead project team verified a sample of 10 percent of all data per municipality. Representatives of the project’s lead team visited all municipalities to oversee the data collection. All study participants signed an informed consent form administered by study personnel, per the regulations established by Colombia’s ethics oversight committee and approved by the ICBF.

### Data Analysis

For the first aim, we analyzed descriptive statistics of the primary study measures. Contingency tables were analyzed to examine the comorbidity of caregiver psychopathology. To test H1, we analyzed bivariate correlations between measures of caregiver psychopathology and child SEC. To supplement this analysis, we examined mean differences in child SEC between caregivers who scored above the cutoff on at least one of the psychopathology measures and caregivers who scored below the cutoff on all psychopathology measures. To probe moderated associations (H2), we analyzed four hierarchical linear regression models: one model that included child sex and caregiver psychopathology and three additional models in which the interaction terms between child sex, caregiver anxiety, PTSD, and depression, respectively, were added. Finally, to examine the moderating role of caregiver resilience in the associations between caregiver psychopathology and child SEC (H3), we conducted hierarchical regression analyses that included the measures of caregiver psychopathology and the interaction between caregiver resilience and psychopathology. In the regression models, continuous scores of caregiver anxiety and PTSD were *z*-standardized before creating the interaction terms. If the data support sex-specific associations between caregiver psychopathology and child SEC (H2), it was planned to run separate regression models for boys and girls. As a complementary analysis assuming comorbidity between the indicators of caregiver psychopathology, we examined the interaction between caregiver resilience and a dichotomous variable indicating whether the caregiver scored above the cutoff score on at least one of the three psychopathology measures.

## Results

### Descriptive Analysis of Caregiver Psychopathology and Child Social-emotional Challenges

The first aim was to describe the extent of Colombian caregivers’ anxiety, depression, and post-traumatic stress symptoms and determine comorbidity. Table 2 shows descriptive statistics of all study measures and the number of individuals scoring above the instrument-specific cutoff scores. Concerning children’s SEC, between 10.8 and 22.8% of the children scored above the ASQ-SE cutoff scores. When comparing caregivers’ scores on the mental health measures, almost half of the sample screened positive for depression, about one-fourth of the sample screened positive for PTSD, and about every-eighth caregiver reported mild-to-moderate and severe symptoms of anxiety. 53.3% of all caregivers (*n* = 604) reported elevated levels of psychopathology above the cutoff on at least one of the three measures. Conversely, 431 caregivers (38.0%) scored below the cutoff on all three measures. Comorbidity was also prevalent in this sample. Regarding sex differences, female and male caregivers did not differ significantly in their levels of psychopathology, and there were no significant mean differences in ASQ-SE scores between boys and girls (Table 2).

**Table 2:** Descriptive statistics and the number of individuals scoring above the instrument-specific cutoff scores

	Total sample					Male children or caregiver				Female children or caregiver			
	*n*	%	*M*	*SD*	α	*n*	%	*M*	*SD*	*n*	*%*	*M*	*SD*
*Child social-emotional challenges*													
24 months^a^ (> 50)	25	11.9	29.43	24.14	0.77	12	5.69	28.90	24.82	12	5.69	29.73	23.73
36 months^b^ (> 59)	94	22.8	42.28	31.71	0.80	46	11.17	41.72	32.90	47	11.41	42.81	30.80
48 months^c^ (> 70)	55	10.8	41.42	31.31	0.81	19	3.73	39.14	29.14	35	6.86	43.37	33.26
Caregiver depression^d^ (yes)	506	44.7				17	44.7			447	45.1		
*Caregiver anxiety*^*e*^			9.48	7.47	0.87			8.26	8.00			9.55	7.46
Mild severity (≤ 17)	924	87.17				31	81.6			823	83		
Mild to moderate severity (18–24)	84	7.93				1	2.6			77	7.8		
Moderate to severe (25–30)	30	2.83				2	5.3			27	2.7		
Severe (> 30)	22	2.08				1	2.6			19	1.9		
*Caregiver PTSD*			25.81	9.86	0.92			25.53	10.38			25.84	9.83
Total score ≥ 30	278	24.5				9	23.7			249	25.1		
*Comorbidity*													
PTSD and anxiety, no depression	13	1.1				0	0			12	1.2		
PTSD and depression, no anxiety	115	10.2				3	7.9			105	10.6		
Depression and anxiety, not PTSD	26	2.3				1	2.6			23	2.3		
PTSD, anxiety and depression	78	6.9				3	7.9			70	7.1		

Instances in which data for male and female caregivers are not consistent with the full sample is due to missing data on child sex (18 children) and caregiver sex (103 caregivers)

^a^*N* = 211

^b^*N* = 412

^c^*N* = 510

^d^Depression was scored as “yes” if participants answered “yes” to one or both questions on the Whooley Depression Screening. 56 participants are missing information on the depression measure

^e^73 participants are missing information on the anxiety measure

^f^96 participants are missing information on the PTSD measure

### Associations Between Child Social-Emotional Challenges, Caregiver Psychopathology, and Caregiver Resilience

In support of H1, we found small, positive correlations between child SEC (ASQ-SE scores) and caregiver psychopathology (Table 3). The strongest correlation was found between child SEC (36-months version) and caregiver depression. The weakest and statistically non-significant correlation was found between child SEC (24-months version) and caregiver anxiety. Further analyses showed that children of caregivers who scored above the cutoff on at least one of the psychopathology measures had significantly higher scores on the ASQ-SE compared to children of caregivers who scored below the cutoff on all three measures. This was found for children assessed using the 24-month (*t* = −3.80, *p* < 0.001, Cohen’s *d* = −0.51), 36-month (*t* = −5.90, *p* < 0.001, Cohen’s *d* = −0.59), 48-month (*t* = −3.48, *p* < 0.001, Cohen’s *d* = −0.33) versions, and the standardized total scores across all age groups (*t* = −7.53, *p* < 0.001, Cohen’s *d* = −0.45). Caregiver resilience (CD-RISC scores) was not significantly associated with caregiver psychopathology or child SEC.

**Table 3:** Correlations between child social-emotional challenges and caregiver psychopathology and resilience

		1	2	3	4	5	6	7	8
1	Child SEC (ASQ-SE total score)		1.00	1.00	1.00	0.25^***^	0.18^***^	0.18^***^	−0.02
2	Child SEC (24 months)					0.25^***^	0.14	0.17^*^	−0.10
3	Child SEC (36 months)					0.32^***^	0.20^***^	0.16^**^	−0.02
4	Child SEC (48 months)					0.19^***^	0.19^***^	0.21^***^	−0.01
5	Depression						0.38^***^	0.33^***^	0.04
6	Anxiety							0.60^***^	0.04
7	PTSD								0.03
8	Resilience^a^								
9	Child age	0.00	0.10	0.06	−0.06	−0.01	0.03	−0.04	0.04
10	Caregiver age	0.00	−0.01	−0.02	0.01	0.07^*^	0.08^*^	0.01	0.02

^a^*N* = 487. *SEC* Social-emotional challenges. The ASQ-SE total score was calculated across age groups (i.e., 24-, 36-, and 48-month-old children) by computing mean-centered scores for each age group, divided by the pooled standard deviation. ***p < 0.001. **p < 0.01. **p < 0.05

### Sex-Specific Associations Between Caregiver Psychopathology and Child Social-Emotional Challenges

To test H2, we analyzed individual differences in children’s ASQ-SE scores as a function of caregiver anxiety, PTSD and depression, and interactions with child sex. The results of the linear regression models are shown in Table 4. Across all models, child caregiver-reported SEC of children were higher for caregivers who screened positive on the depression measure. The association between caregiver anxiety and child SEC was not statistically significant. A larger degree of caregiver-reported PTSD symptoms were associated with higher levels of child SEC (Model 1). The interaction between caregiver PTSD and child sex in predicting child SEC was not statistically significant (Model 3). The interaction between caregiver depression and child sex was negative and statistically significant (Model 4). This finding indicates that caregiver depression was more strongly associated with SEC of boys (*B* = 0.56, *p* < 0.001, 95% CI 0.37, 0.74) than girls (*B* = 0.22, *p* = 0.02, 95% CI 0.04, 0.40). However, it is essential to note this association was also statistically significant for girls. On average, boys whose caregivers screened positive for depression showed higher levels of SEC (*M* = 0.25) than boys whose caregivers screened negative for depression (*M* = −0.31). As implied by the significant interaction term, for girls, the difference between girls whose caregivers screened positive for depression (*M* = 0.17) and girls whose caregivers screened negative for depression (*M* = −0.05) was less pronounced compared to boys. These findings support H2 and suggest that child sex moderates the association between caregiver psychopathology (i.e., depressive symptoms) and child SEC. However, *R*^2^ values were rather small with between 6.5 and 8% of the total variance accounted for by the predictor variables. This finding implies associations between child SEC and other, unobserved variables.

**Table 4:** Linear (OLS) regression models predicting child social-emotional challenges based on caregiver psychopathology and interactions with child sex

	Model 1	Model 2	Model 3	Model 4
	*B* (95% CI)	*B* (95% CI)	*B* (95% CI)	*B* (95% CI)
Intercept	−0.53 (−0.72, −0.35)	−0.48 (−0.71, -0.25)	−0.29 (−0.43, −0.15)	−0.63 (−0.83, −0.43)
Child sex	0.10 (−0.03, 0.22)	0.10 (−0.03, 0.22)	0.10 (−0.03, 0.22)	0.26** (0.09, 0.43)
Caregiver anxiety	0.01 (−0.00, 0.02)	0.01 (−0.00, 0.02)	0.01 (−0.00, 0.02)	0.01 (−0.00, 0.02)
Caregiver PTSD	0.01* (0.02, 0.00)	0.01* (0.00, 0.02)	0.01 (−0.01, 0.02)	0.01* (0.00, 0.02)
Caregiver depression ^a^	0.38*** (0.25, 0.52)	0.38*** (0.25, 0.52)	0.39*** (−25, 0.52)	0.56*** (0.37, 0.74)
Interaction: Caregiver anxiety by child sex		−0.01 (−0.02, 0.01)		
Interaction: Caregiver PTSD by child sex			0.01 (−0.01, 0.02)	
Interaction: Caregiver depression by child sex				−0.34** (−0.59, −0.09)

*F*	18.57, *p* < 0.001	14.97, *p* < 0.001	15.02, *p* < 0.001	16.35, *p* < 0.001
*R*^2^	0.065	0.07	0.07	0.08
Δ*R*^2^		0.0006	0.0008	0.0065**

***p < 0.001

**p < 0.01. *p < 0.05

^a^Categorical variable (0 = negative screening, 1 = positive screening)

### Caregiver Resilience Moderates the Association Between Caregiver Psychopathology and Child Social-emotional Challenges

**Fig. 1:**
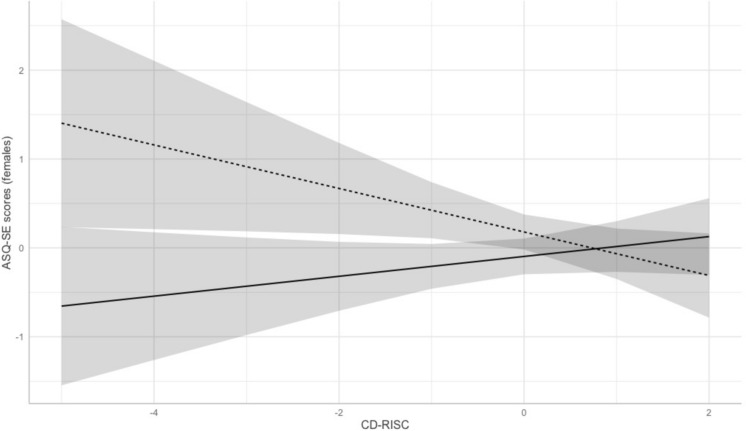
Interaction between caregiver resilience (CD-RISC scores, x-axis) and social-emotional challenges (ASQ-SE scores, y-axis) of female children. The dotted line shows the slope of the association between caregiver resilience and female children’s social-emotional challenges for caregivers who scored above the cutoff on at least one of the psychopathology measures. The bold line reflects the slope of the association between caregiver resilience and female children’s social-emotional challenges for caregivers who scored below the cutoff on all psychopathology measures

Finally, to test H3, we conducted a series of moderated regression analyses to probe for interactions between caregiver psychopathology and resilience predicting child SEC. Separate models were tested for anxiety, PTSD, and depression in the complete sample of caregivers with available CD-RISC data. There were no statistically significant interaction terms in the estimated models (Supplemental Table 1). Given the significant interaction between caregiver depression and child sex, we ran separate models for boys and girls to test the interactions between caregiver psychopathology and caregiver resilience. None of the scores of the three measures of caregiver psychopathology interacted with caregiver resilience to account for child SEC. However, given the extent of comorbidity, we conducted a complementary analysis based on a dichotomous variable indicating whether the caregiver scored above the cutoff score on at least one of the three psychopathology measures. In the entire sample, as well as for boys, the interaction between this variable and resilience was not statistically significant. For girls, however, caregiver resilience moderated the association between the dichotomous variable created to measure caregiver psychopathology and child SEC, *B* = −0.36, *p* = 0.016, *F*(3, 234) = 3.17, *p* = 0.025, *R*^2^ = 0.039. Figure 1 illustrates this interaction. The slope analysis showed that there was a positive but statistically non-significant association between caregiver resilience and child SEC for caregivers who scored below the cutoff on all psychopathology measures, *B* = 0.11, *p* = 0.22. In contrast, there was a negative, statistically significant association between caregiver resilience and child SEC for caregivers who scored above the cutoff on at least one of the psychopathology measures, *B* = −0.25, *p* = 0.04. Together, these analyses partially support H3, but the moderating role of caregiver resilience was specific to girls, as no statistically significant interactions were identified among boys.

## Discussion

The present study sought to contribute to the emerging literature on the mental health of young children and their caregivers in areas affected by armed conflict and displacement in Colombia. Our first aim was to quantify the extent of anxiety, depression, and symptoms of post-traumatic stress of Colombian caregivers in areas exposed to armed conflict and the social-emotional challenges of children. We found substantial levels of psychopathology that are above the average of the general population in Colombia [15], and related studies that examined NMHS data in Colombia and found prevalence rates of PTSD and any mental health disorder of 12.3 and 11%, respectively [26], and 15.9% lifetime prevalence of any psychiatric disorder in displaced adults [38]. Reasons for this discrepancy could be the higher rates of self-reported displacement and victimization by the armed conflict in this sample (44 and 20.4%, respectively) compared with about 15% reported in the literature [39]. The higher rates were, however, expected given the purposive sampling from areas with the highest levels of armed conflict. For children, up to 22.8% of the children (i.e., the children whose caregiver provided information using the ASQ-SE for 36-month-olds) scored above the age-specific cutoff on the ASQ-SE. Few studies have used the ASQ-SE in South American countries. One study examined the psychometric properties of the Spanish version of the ASQ-SE in a nationally representative sample of children aged 3–65 months (*M* = 36.7 months) in Uruguay [1]. The authors reported mean scores of 25.9, 38.7, and 39.5 for 24-, 36-, and 48-month-olds, respectively, which were all lower compared to children in this study. However, the children in the present Colombian sample scored overall lower compared to individuals in the seminal ASQ-SE study, which reported means of 35.4, 49.9, and 55.7 for 24-, 36-, and 48-month-olds, respectively [37]. Our study is among the first to contribute comparative data on children’s SEC in Colombia using the ASQ-SE.

The second aim was to examine the associations between caregiver psychopathology and the SEC of their child. Our findings demonstrate that higher levels of caregiver psychopathology showed small but statistically significant associations with higher levels of child SEC, particularly in the context of social violence/armed conflict. These findings align with results from a study from South Africa that showed positive associations between comorbid prenatal depression and trait anxiety in mothers with child social-emotional problems at age 3 [36]. These findings also align with Flink et al.’s [13] study of internally displaced preschool children in Colombia that identified associations between caregiver mental health and children’s scores above the cut-off on the CBCL. Collectively, our findings highlight the importance of caregiver mental health as a potential risk factor likely due to stress related to exposure to violence, potential consequences of intergenerational trauma, and the transition pathways of ACEs across generations [32]. Specifically, improvements in caregiver mental health and parenting-related stress have demonstrated the potential to improve children's developmental outcomes in light of adverse experiences [40]. Our findings add to this literature by highlighting the need to support caregivers exposed to armed conflict and who report symptoms of psychopathology.

The exact biopsychosocial mechanisms that may account for the associations between caregiver psychopathology and child SEC are understudied, particularly for younger children in Colombia. Although this study did not examine the mechanisms by which caregiver psychopathology is related to child SEC, prior literature suggests that caregiver psychopathology may be related to impaired child-caregiver relational health, lack of responsive parenting, harsh or ineffective parenting practices (e.g., [12, 35]), which have been associated with socio-emotional challenges and behavior problems. Although there are limited studies that examine these effects in caregiver-child dyads living in the context of armed conflict, it is possible that the stress related to armed conflict and other psychosocial stressors directly impacts caregiving practices and/or directly impacts caregiver psychopathology, which in turn affects caregiver practices and leads to child SEC. More studies are needed to understand this mechanism to develop targeted interventions supporting caregivers living in armed conflict. Studies from the U.S. and Canada suggest that responsive parenting and higher quality childcare settings may be possible buffers to caregiver psychopathology and child SEC [5]. In Colombia, more longitudinal research is needed to examine the potential buffering role of quality ECD services against the negative impact ofcaregiver depression.

Concerning the third aim, our data suggest that child sex moderates the relationship between caregiver psychopathology and child SEC. Specifically, boys are more vulnerable to the effects of caregiver depression on their SEC. These findings complement other findings in the literature that demonstrate stronger associations among boys between maternal PTSD, anxiety, and depressive symptoms with the problem behaviors of young children [28, 31], particularly internalizing behaviors [23]. Sex differences may result from bidirectional associations between maternal parenting behavior and child characteristics such that boys, on average, exhibit more externalizing behavior problems than girls and, therefore, tend to elicit more harsh behavior from their parents (e.g., [48]). Another possible interpretation is that caregivers with depressive symptoms may identify and report more problems in boys as they can find these behaviors more challenging to manage when feeling emotionally affected. Whether such bidirectional associations generalize to caregivers with adverse, potentially traumatic experiences due to exposure to armed conflict remains to be examined.

Finally, the fourth aim was to probe whether caregiver resilience moderates the association between caregiver psychopathology and child SEC. Our findings showed that resilience buffered the negative effect of caregiver psychopathology on child SEC, but this interaction was specific to girls. However, this effect only emerged when caregiver psychopathology was scored dichotomously (i.e., scoring above the cut-off on at least one of the measures of anxiety, depression, or PTSD). Thus, there is only limited support for the moderating role of resilience in this sample. Possible explanations for this finding stem from how resilience was measured in this study. The original 25-item version of the CD-RISC scale includes items that measure, for instance, personal competence, tolerance of negative affect, perceptions of control, and positive acceptance of change and secure relationships. When experiencing symptoms of psychopathology, a caregiver’s belief that coping with stress strengthens them and that they can handle unpleasant feelings may positively impact parenting behavior. Resilience has been found to correlate with responsive parenting, which has been found to buffer the impact of maternal PTSD on young children [19]. This has potential indirect effects on reduced levels of conflict between parents and children and reduced caregiver burden for children as young as three years of age [34]. Thus, it is plausible that resilience may enhance responsive parenting and family functioning, which are positively associated with child adjustment.

Caregiver resilience, as measured in this study by CD-RISC, focuses on individual intrapersonal factors that enable caregivers to withstand and adapt to adversities. However, this approach may not fully capture the complex interplay between personal resilience and systemic issues such as ongoing violence and displacement. While our findings indicate that caregiver resilience was not significantly correlated with caregiver psychopathology or child social and emotional challenges, this does not negate the importance of resilience. Instead, it highlights the necessity of a nuanced understanding that differentiates between individual resilience and the broader socio-political environment. Critiques of resilience theory, such as those presented by Grant et al. [18] and McCrae et al. [27], underscore the need for future research to integrate systemic factors into the study of resilience. This study, being part of a larger project aimed at promoting caregiver resilience, acknowledges these complexities and aims to refine our understanding of resilience in such challenging contexts. Future directions should include addressing these critiques by incorporating systemic violence issues and exploring how they intersect with intrapersonal resilience factors.

Furthermore, close relationships with competent and caring adults in the family and community are vital features associated with resilient functioning [6]. Thus, the belief of the Colombian caregivers in our sample that close and secure relationships are present in one’s life may also encourage them to seek support in raising their young child, which may positively impact the child’s social-emotional development. Such sources of psychosocial support are significant for caregivers with PTSD, as traumatized parents were found to be emotionally less available and to perceive their children more negatively than parents without symptoms of PTSD in a literature review of 72 studies on traumatized parents [42].

### Limitations and Future Directions

The present study utilized data from a large-scale epidemiological survey on adult mental health in Colombia, allowing for robust inferences on the hypothesized correlations. Nevertheless, several limitations must be acknowledged. First, the data were gathered almost ten years ago, in 2015. However, we consider this study’s results to be highly relevant today. There is a scarcity of literature that explores the relationship presented here within a broad, diverse population that has been affected by armed conflict. This gap underscores the importance of our findings and their continued relevance to understanding and addressing the mental health and resilience of caregivers in conflict-affected settings. Furthermore, the data we have gathered have been instrumental in refining evaluation processes across the country. Specifically, they have been crucial in shaping the national mental health policy and guiding targeted actions to support the mental health of populations affected by armed conflict. Currently, there is an ongoing debate both in Colombia and throughout Latin America regarding the use of psychosocial development scales. This discussion is closely linked to the implications of mental health of parents and parental figures for children's development. Our findings highlight the need for a holistic approach that considers the mental health of parents and caregivers as a critical factor in the psychosocial development of children. This perspective is now being integrated into national strategies, ensuring that both child and parental mental health are addressed concurrently, thereby enhancing the effectiveness of interventions and support programs.

Second, the cross-sectional design precludes the analysis of causal relationships. Future studies should aim to assess children and their caregivers multiple times, preferably at least three, to examine changes in child SEC, caregiver mental health, and resilience over time. Such a study would also yield important information about the correlations between the trajectories of children and their caregivers. Third, relying only on one informant to report on caregiver psychopathology, resilience, and child SEC may introduce a mono-method bias. Furthermore, using short screeners (as opposed to full questionnaires and diagnostic interviews) to assess psychopathology increases the likelihood of false-positives and overestimation of caregiver psychopathology in this sample. Future studies, should consider multi-informant and multi-method methods to assess psychopathology and resilience.

Fourth, resilience data were available only for a smaller subsample of caregivers. This subsample was also slightly biased in that caregivers without resilience data reported higher levels of psychopathology. Two potential selection biases are evident. Caregivers may have opted not to participate in the study due to fear of, or being exposed to, the armed conflict. For some caregivers, the willingness to talk about painful experiences and situations depended partly on their perception of the availability of strong community support networks. Thus, a larger, representative sample would be needed to solidify the present study’s findings. Given the high interdependence between people in the Colombian culture and a high tendency toward solidarity, Colombians have sought and strengthened systems of social support over the past decades, such as the kinship social net (i.e., caregiving and in-kind help from consanguine or nonconsanguine others; [41]). Therefore, it would also be necessary to better contextualize resilience in Colombia and include social-ecological indicators of resilience (e.g., feelings of belonging to one's community, social support available in the extended social network; [24]). There is also a need to move beyond the individual caregiver and their well-being to better understand mental health outcomes and resilience of the family unit, including the elderly and community support groups in the municipalities. Future research should consider characterizing the experienced adverse circumstances and how they predict resilient developmental pathways of caregivers and their children in Colombia.

Fifth, the data analyzed in this study included only a few male caregivers and a few older caregivers. The moderating role of child sex may also extend to caregivers other than the mother, and gender of the child may also play a role in development. Ramchandani and Psychogiou [33] synthesized research suggesting that the associations between paternal psychiatric disorders and children’s social and emotional difficulties are greater for boys than for girls. Thus, including fathers, and other caregivers, and the role of gender both of caregiver and children in parenting and mental health research in Colombia is crucial. Particularly as research suggest that fathers and other caregivers desire to participate more at home and to be more involved in their children’s lives [4]. Therefore, future research should aim to contextualize further the meaning of caregiving and caregiver mental health utilizing models more specific to Colombia. Finally, in Colombia, mental health may be perceived as shameful, stigmatized, and often not verbalized, particularly among men [17]. Moreover, the risk faced by caregivers when reporting that they are victims of the armed conflict is a limitation not only for access to health services but also for being able to refer to painful experiences and situations and to be able to seek psychosocial support.

## Conclusions

The previous literature in Colombia focuses on only one or two, but not all, of the following constructs: caregiver mental health, child social-emotional development, resilience, and the armed conflict in Colombia. The present study is unique in examining the relationship between these factors. Thus, these findings have implications for providing psychosocial support, particularly for mothers with depressive symptoms and with a particular emphasis on parent–child interactions. The psychopathology prevalence identified in this study necessitates accelerating the integration of interventions that improve caregiver mental health into early childhood interventions. Psychosocial support should include structured training to build positive relationships with children and interaction with peers to develop resilience in coping with disruptive events. Such pivotal work is underway in Colombia. For instance, our recent study [16] found that the resilience of caregivers significantly improved throughout participating in a program called the “Commigo, Contigo y Con Todos” (3C), which includes a combination of skills-building (e.g., parenting practices, life skills, stress management) and psychotherapy (e.g., third-generation cognitive behavioral techniques). It is vital to expand these efforts to ensure caregivers in armed conflict have access to support and resources and that they can learn effective coping strategies to ensure nurturing care.

## Summary

This study presents a comprehensive investigation of the mental health and resilience of caregivers in conflict-affected areas of Colombia, focusing on the impacts of these factors on early childhood development (ECD). Colombia, with over 50 years of civil conflict, presents a unique case for examining these impacts. The lingering effects of conflict have exposed many young children to developmental risks such as poverty, violence, and displacement. The present research suggests a strong link between caregiver mental health and child adjustment. Caregiver psychopathology, particularly depression and anxiety, has been shown to predict problem behaviors in young children, with boys being more vulnerable than girls. This study emphasizes the critical need for supporting caregiver mental health and resilience to promote better developmental outcomes for children in conflict-affected settings. It underscores the importance of empirical evidence to inform effective interventions and humanitarian responses.

## Supplementary Information

Below is the link to the electronic supplementary material.

Supplementary file1 (DOCX 51 KB)

## Data Availability

Data can be requested from the corresponding author.
