# Supplementary material for: Caregiver Psychopathology, Resilience, and Their Associations with Social-Emotional Challenges of Young Children Affected by Armed Conflict in Colombia
Source: Child Psychiatry Hum Dev. 2024 Nov 22;57(4):1225–37. doi: 10.1007/s10578-024-01787-y (PMC13437763; doi:10.1007/s10578-024-01787-y)
Supplement: Supplementary file 1 [file 10578_2024_1787_MOESM1_ESM.docx]

Supplemental Table 1

*Linear (OLS) regression models predicting child social-emotional challenges based on caregiver psychopathology and interactions with caregiver resilience*

|  | Model 1 | Model 2 | Model 3 |
| --- | --- | --- | --- |
|  | *B* (95%-CI) | *B* (95%-CI) | *B* (95%-CI) |
| Intercept | -0.03 (-0.17, 0.11) | -0.06 (-0.20, 0.08) | -0.25^**^ (-0.41, -0.08) |
| Child sex | 0.06 (-0.14, 0.25) | 0.11 (-0.09, 0.30) | 0.09 (-0.10, 0.28) |
| Caregiver anxiety | 0.21^***^ (-0.14, 0.25) |  |  |
| Caregiver PTSD |  | 0.17^***^ (0.07, 0.28) |  |
| Caregiver depression ^a^ |  |  | 0.45^***^ (0.25, 0.64) |
| Caregiver resilience | -0.04 (-0.14, 0.06) |  |  |
| Interaction: Caregiver anxiety by caregiver resilience | -0.09 (-0.19, 0.02) |  |  |
| Interaction: Caregiver PTSD by caregiver resilience |  | -0.10 (-0.21, 0.01) |  |
| Interaction: Caregiver depression by caregiver resilience |  |  | -0.12 (-0.32, 0.07) |
| *F* | 4.85^***^ | 4.14^**^ |  |
| *R*^2^ | 0.04 | 0.05 |  |

*Notes*. ^a^ Categorical variable (0 = negative screening, 1 = positive screening). *** *p* < .001. ** *p* < .01. * *p* < .05.

Supplemental Table 2

*Demographic characteristics of the sample of caregivers with available resilience (CD-RISC) data (n = 487)*

|  | Full sample (*N* = 1,133) | | | | Caregivers with available resilience (CD-RISC) data  (*n* = 487) | | | |
| --- | --- | --- | --- | --- | --- | --- | --- | --- |
|  | *n* | % | *M (SD)* | Min, Max | *n* | % | *M (SD)* | Min, Max |
| **Child** | | | | | | | | |
| Age (months) | 1133 |  | 39.08 (8.99) | 21, 53 | 487 |  | 39.56 (8.99) | 21, 53 |
| Child SEC (ASQ-SE score, 24 months) | 211 | 18.62 | 29.43 (24.14) | 0, 165 | 87 | 17.86 | 27.36 (17.81) | 5, 90 |
| Child SEC (ASQ-SE score, 36 months) | 412 | 36.36 | 42.28 (31.71) | 5, 200 | 166 | 34.09 | 44.94 (34.63) | 5, 200 |
| Child SEC (ASQ-SE score, 48 month) | 510 | 45.01 | 41.42 (31.31) | 5, 195 | 234 | 48.05 | 39.38 (31.15) | 5, 190 |
| Sex |  |  |  |  |  |  |  |  |
| Male | 535 | 47.2 |  |  | 225 | 46.2 |  |  |
| Female | 580 | 51.2 |  |  | 252 | 51.7 |  |  |
| Missing | 18 | 1.6 |  |  | 10 | 2.1 |  |  |
| Birth order |  |  |  |  |  |  |  |  |
| First-born | 1109 | 97.9 |  |  | 481 | 98.8 |  |  |
| Second-born | 24 | 2.1 |  |  | 6 | 1.2 |  |  |
| Disability (parent report) |  |  |  |  |  |  |  |  |
| Yes | 389 | 34.3 |  |  | 141 | 29 |  |  |
| No | 687 | 60.6 |  |  | 334 | 68.6 |  |  |
| Missing | 57 | 5 |  |  | 12 | 2.5 |  |  |
| Victim of forced displacement |  |  |  |  |  |  |  |  |
| Yes | 500 | 44.1 |  |  | 253 | 52 |  |  |
| No | 575 | 50.8 |  |  | 214 | 43.9 |  |  |
| Missing | 58 | 5.1 |  |  | 20 | 4.1 |  |  |
| Victim of armed conflict |  |  |  |  |  |  |  |  |
| Yes | 231 | 20.4 |  |  | 104 | 21.4 |  |  |
| No | 845 | 74.6 |  |  | 371 | 76.2 |  |  |
| Missing | 57 | 5 |  |  | 12 | 2.5 |  |  |
| Municipality |  |  |  |  |  |  |  |  |
| Albania | 8 | 0.7 |  |  | 3 | 0.6 |  |  |
| Buenaventura | 75 | 6.6 |  |  | 12 | 2.5 |  |  |
| El Tambo | 61 | 5.4 |  |  | 32 | 6.6 |  |  |
| Guapi | 77 | 6.8 |  |  | 22 | 4.5 |  |  |
| Istmina | 92 | 8.1 |  |  | 24 | 4.9 |  |  |
| Maicao | 20 | 1.8 |  |  | 15 | 3.1 |  |  |
| Medellin | 75 | 6.6 |  |  | 17 | 3.5 |  |  |
| Nariño | 1 | 0.1 |  |  |  |  |  |  |
| Necocli | 95 | 8.4 |  |  | 33 | 6.8 |  |  |
| Pasto | 64 | 5.6 |  |  | 27 | 5.5 |  |  |
| Popayán | 1 | 0.1 |  |  | 1 | 0.2 |  |  |
| San Vincente del Caguán | 99 | 8.7 |  |  | 22 | 4.5 |  |  |
| Sincelejo | 78 | 6.9 |  |  | 36 | 7.4 |  |  |
| Soledad | 57 | 5.0 |  |  | 43 | 8.8 |  |  |
| Tame | 86 | 7.6 |  |  | 74 | 15.2 |  |  |
| Totumo | 1 | 0.1 |  |  |  |  |  |  |
| Tumaco | 66 | 5.8 |  |  | 26 | 5.3 |  |  |
| Turbo | 166 | 14.7 |  |  | 95 | 19.5 |  |  |
| Missing | 11 | 1.0 |  |  | 5 | 1.0 |  |  |
| **Caregiver** | | | | | | | | |
| Age (years) | 1041 |  | 29.39 (8.32) | 16, 75 | 460 |  | 29.82 (8.53) | 16, 65 |
| Missing | 92 | 8.12 |  |  | 27 | 5.5 |  |  |
| Sex |  |  |  |  |  |  |  |  |
| Male | 38 | 3.4 |  |  | 12 | 2.5 |  |  |
| Female | 992 | 87.6 |  |  | 440 | 90.3 |  |  |
| Missing | 103 | 9.1 |  |  | 35 | 7.2 |  |  |
| Victim of forced displacement |  |  |  |  |  |  |  |  |
| Yes | 471 | 41.6 |  |  | 243 | 49.9 |  |  |
| No | 584 | 51.5 |  |  | 232 | 47.6 |  |  |
| Missing | 78 | 6.9 |  |  | 12 | 2.5 |  |  |
| Victim of armed conflict |  |  |  |  |  |  |  |  |
| Yes | 395 | 34.9 |  |  | 104 | 21.4 |  |  |
| No | 681 | 60.1 |  |  | 371 | 76.2 |  |  |
| Missing | 57 | 5.0 |  |  | 12 | 2.5 |  |  |
| Depression (screened positive) | 506 | 44.7 |  |  | 197 | 40.5 |  |  |
| Anxiety (HAM-A total score) | 1060 | 93.56 | 9.48 (7.47) | 0, 46 | 463 | 95.1 | 8.88 (7.10) | 0, 46 |
| PTSD (PCL-C total score) | 1037 | 91.53 | 25.81 (9.86) | 17, 74 | 459 | 94.3 | 24.82 (9.36) | 17, 74 |

*Notes*. SEC = social-emotional challenges.
